# The associations of osteoporosis and possible sarcopenia with disability, nutrition, and cognition in community-dwelling older adults

**DOI:** 10.1186/s12877-023-04431-x

**Published:** 2023-11-10

**Authors:** Yin-Yi Chou, Cheng-Fu Lin, Yu-Shan Lee, Shuo‑Chun Weng, Fu-Hsuan Kuo, Chiann-Yi Hsu, Shih-Yi Lin

**Affiliations:** 1 Center for Geriatrics & Gerontology, Taichung Veterans General Hospital, Taichung, 40705 Taiwan; 2 Division of Allergy, Immunology and Rheumatology, Department of Internal Medicine, Taichung Veterans General Hospital, Taichung, 40705 Taiwan; 3 Division of Occupational Medicine, Department of Emergency, Taichung Veterans General Hospital, Taichung, 40705 Taiwan; 4 Geriatrics and Gerontology Research Center, College of Medicine, National Chung Hsing University, Taichung, 40200 Taiwan; 5 Department of Post-Baccalaureate Medicine, College of Medicine, National Chung Hsing University, Taichung, 40200 Taiwan; 6 Division of Neurology, Taichung Veterans General Hospital, Taichung, 40705 Taiwan; 7 Division of Nephrology, Department of Internal Medicine, Taichung Veterans General Hospital, Taichung, 40705 Taiwan; 8 Institute of Clinical Medicine, School of Medicine, National Yang Ming Chiao Tung University, Taipei, 11221 Taiwan; 9 Biostatistics Task Force of Taichung Veterans General Hospital, Taichung, 40705 Taiwan; 10 Division of Endocrinology and Metabolism, Department of Internal Medicine, Taichung Veterans General Hospital, Taichung, 40705 Taiwan

**Keywords:** Osteosarcopenia, Outpatient, Community-dwelling older adults, Comprehensive geriatric assessment, Cognitive impairment

## Abstract

**Background:**

Osteoporosis and sarcopenia, respectively, have detrimental impact on health, and combination of both conditions, termed osteosarcopenia, is becoming an increasingly important disorder in older adults as populations age. This study aimed to explore the relationship between osteoporosis and possible sarcopenia and their joint effect on physical performance, nutritional status, and cognition in community-dwelling older adults.

**Methods:**

This study was conducted at a medical center in Taiwan, which included the adjacent community care station. The participants were recruited through regular activities at the community care station between January 01, 2015 and February 28, 2022. During the study period, dual-energy X-ray absorptiometry and comprehensive geriatric assessment consisting of comorbidity burden, functional status, cognition, mood, and nutritional status were performed during the study period. Possible sarcopenia was identified utilizing the criteria set by the Asian Working Group on Sarcopenia in 2019 using the criteria of low muscle strength alone, and osteoporosis was defined by the World Health Organization criteria. Accordingly, the study subjects were divided into four groups: normal, only osteoporosis, only possible sarcopenia, and possible osteosarcopenia.

**Results:**

There were 337 participants (68.6% female) with a median age of 78.0 years (interquartile range: 71.0–85.0 y/o). According to the clinical definition of osteosarcopenia, 78 participants were normal, 69 participants showed possible sarcopenia, 61 participants had osteoporosis, and 129 had osteoporosis with possible sarcopenia. Among the four groups, the prevalence rates of chronic illness, functional capacity, physical performance, cognitive impairment, and malnutrition revealed statistically significant differences. Using logistic regression analysis after adjusting for the other covariates, osteoporosis with possible sarcopenia was associated with an increased odds ratio of cognitive impairment.

**Conclusions:**

The findings suggest that compared to osteoporosis or possible sarcopenia alone, osteoporosis with possible sarcopenia was more likely to be associated with cognitive impairment. Early identification and targeted interventions for cognitive impairment in older adults with osteosarcopenia may be valuable in maintaining cognitive well-being and overall quality of life.

## Background

 Populations worldwide have aged dramatically over recent decades, resulting in increased numbers of older adults with multimorbidity, a trend which has become a major public health issue [1]. Indeed, the challenges faced by health systems in providing equitable access to primary care services are concerning. Vulnerable populations, including older individuals, those facing early mortality risks, and those at risk of hospitalization, functional decline, or nursing home placement, are particularly affected [2]. Ensuring that these vulnerable groups receive adequate and appropriate healthcare services is essential for promoting overall well-being and reducing disparities in health outcomes [3–6]. Healthy aging depends on the ability to maintain the reserve capacity of multiple physiological systems [7]. The comprehensive geriatric assessment (CGA) is a multidisciplinary diagnostic and treatment process that determines a patient’s medical, psychological, and functional capabilities [3–6]. The underlying concept involves identifying individuals who are at high risk of complications and adverse outcomes early so that patient-centered goals can be set, which would allow better allocation of resources and enable a multidisciplinary team to develop a more appropriate treatment [3–6]. The components of CGA are functional status, fall risk, cognition, mood and emotional status, nutritional status, comorbidities and polypharmacy, social support, financial concerns, goals of care, and advanced care planning [3–6].

The musculoskeletal system is composed of bone and muscle and not only enables human ambulation, but also serves as a major metabolic storage site. Age-related deterioration of the musculoskeletal system is common [7]. Osteopenia/osteoporosis is a condition of low bone mass and deterioration of bone tissue leading to fragility fractures, as well as decreased quality of life and mortality [8]. According to the World Health Organization (WHO) criteria, patients with T-scores of bone mineral density (BMD) below − 1 and − 2.5 are categorized as having osteopenia and osteoporosis, respectively [9]. Sarcopenia is a skeletal muscle disease that presents with muscle mass loss and weakness, which are associated with physical decline. The European Working Group on Sarcopenia in Older People revised the practical clinical definition and consensus diagnostic criteria in 2018 [10]. Then in 2019, the Asian Working Group for Sarcopenia (AWGS) also updated the specified cutoffs [11]. A sarcopenia diagnosis is confirmed by the presence of low muscle quantity or quality measured by dual-energy X-ray absorptiometry (DEXA) or bioelectrical impedance analysis (BIA). Possible sarcopenia is identified by low handgrip strength (HGS) or low physical performance assessed by the five times sit-to-stand test. If the criteria of low muscle mass, low handgrip strength, and low physical performance are all met, a patient is considered to have severe sarcopenia [10, 11]. The combination of osteopenia/osteoporosis and sarcopenia was first discussed in 2009 [12] and since 2017 this has been termed osteosarcopenia [13] due to the similar risk factors that include genetics, endocrine function, and mechanical factors. Osteosarcopenia has a substantial cumulative and synergistic impact, resulting in physical decline, malnutrition, and cognitive impairment. This interplay significantly contributes to exacerbation of diverse health outcomes, including an elevated risk of falls, heightened frailty, and increased mortality rates [14].

As osteosarcopenia affects multiple aspects of health in older adults, CGA can be used to evaluate the effects of osteosarcopenia on several geriatric disorders. This study aimed to investigate the correlation between osteoporosis/osteopenia and possible sarcopenia was defined according to AWGS2019 using the criteria of low muscle strength alone, as well as the potential synergistic effects among disability, cognition, and nutrition in the targeted population. Moreover, this research endeavored to identify and analyze the salient risk factors for several components in the CGA among these individuals, in order to gain comprehensive insights into the underlying relationships and mechanisms.

## Methods

### Study design

This study was conducted at a medical center and its adjacent community care station in Taiwan in 2023 and used retrospective data for the period January 01, 2015 to February 28, 2022. The study was approved by the Institutional Review Board of the medical center (IRB no: CE22167A) and all methods were carried out in accordance with the approved study protocol under the standard regulations and the Declaration of Helsinki.

### Participants

Community-dwelling older adults aged 65 years and above were recruited through regular activities at their adjacent community care station. At first, CGA was performed and then patients who were referred to the medical center for further management, such as diagnosis of osteopenia/osteoporosis, rehabilitation program, or nutrition education within one year, were consecutively enrolled. The exclusion criteria were participants refused evaluation, or they could not complete the whole study period for any reason. Initially, 406 participants were enrolled in the study. Of these, 69 participants who just received CGA without further management were excluded. Finally, 337 participants were included in the analysis.

### Diagnosis of sarcopenia, osteoporosis, and osteosarcopenia

Upper extremity mobility was evaluated by handgrip strength using a handheld dynamometer (Smedley’s Dynamometer, TTM, Tokyo, Japan), with a value less than 28 kg indicating low muscle strength for men and 18 kg for women [11]. Furthermore, low hand grip strength was defined as possible sarcopenia. Lower extremity mobility was evaluated by the 6-meter walking speed (6MWS) test, with a value less than 1 m/s indicating low physical performance [11]. The BMD of the lumbar spine (L2–L4), femoral neck, and total hip was assessed using DEXA (DXA; Lunar iDXA, GE Healthcare, Chicago, IL, USA). If BMD was below − 1 or -2.5 for at least one of these three measurements, a diagnosis of osteopenia or osteoporosis, respectively, was made, in accordance with the WHO criteria [9]. Possible osteosarcopenia was defined as the coexistence of osteoporosis and possible sarcopenia.

### Geriatric assessment

Basic personal information and medical records of all enrolled participants were reviewed, including age, gender, body height, body weight, body mass index (BMI), educational level, religion, personal habit (cigarette, alcohol, and betel nut use), marital status, family relationship, social support, financial concerns, disease burden measured by Charlson comorbidity index [15], and medications. The CGA was administered initially by a well-trained nurse while the participants received community care service regularly. In brief, the CGA measured functional capacity, cognition, mood, and nutrition. Functional capacity was assessed by the Barthel index (BI) of activities of daily living (ADL), including ten basic activities, and the range of BI was 0 to 100, with lower values on the scale meaning greater dependence on others [16]. The BI score of 60 points or lower was considered an ADL disability [16]. The Lawton Instrumental ADL (IADL) scale comprises eight domains related to performing tasks and is scored on a scale of 0 to 8, with lower scores indicating poorer ability [17]. To measure both static and dynamic balance, the timed up and go (TUG) test was examined and the examination required the participants to rise from a chair, walk straight for 3 m, turn around 180 degrees, go back to the chair, and then sit finally. If they took longer than 30 s to complete the test, it meant the participants had a high risk for falling and required a gait aid [18]. Cognition was measured by the mini-mental state examination (MMSE), consisting of orientation, registration, attention/calculation, recall, language, repetition, and complex commands, and the score ranged from 0 to 30. A score of MMSE less than or equal to 24 indicated cognitive impairment if the participant was literate or less than or equal to 13 if illiterate [19]. Mood was evaluated by the five-item geriatric depression scale (GDS-5) and depressive symptoms were defined as a GDS-5 score greater or equal to 2 [20]. Nutritional status was assessed by the mini-nutritional assessment-short form (MNA-SF), consisting of six questions, and the score ranged from 0 to 14. A score less than 12 indicated a risk of malnutrition [21].

### Statistical analyses

Non-parametric tests were used because the data did not adhere to a normal distribution. Continuous variables were expressed as median and interquartile range (IQR, 25-75%). Categorical data were expressed as number and percentage. The significance of the difference between groups was assessed using the Mann-Whitney U test, the Kruskal-Wallis test for continuous variables, and the Chi-Square test for categorical variables. Logistic regression models were used to determine the association between possible sarcopenia, osteoporosis, and possible osteosarcopenia with physical and cognitive dysfunction and malnutrition. The results are shown in terms of an odds ratio. We included the known factors that may modify the effect of this association and that have been previously described in the literature. The variables included in the final multivariate analysis were those significantly related with dichotomous outcomes in univariate analysis. Statistical analyses were performed using SPSS version 22.0 (SPSS Inc., Chicago, IL, USA). A two-tailed p-value of < 0.05 was considered statistically significant. Due to the exploratory nature of this study, no adjustment of p values was made for multiple comparisons.

## Results

The basic characteristics and CGA of the participants are shown in Table 1. In total, the median age was 78.0 (IQR 71.0–85.0) years old with a female-to-male ratio of 68.5:31.5. The median height, weight, and BMI were 155.4 (IQR 151.1-161.2) cm, 58.0 (IQR 51.0-65.1) kg, and 23.5 (IQR 21.1–25.9). The BI was 95.0 (85.0-100.0) and 9.5% of the participants had ADL disability. HGS was 19.0 (14.6–24.3) kg and 58.8% had low muscle strength. MMSE score was 26.0 (20.0–29.0) and 28.5% showed cognitive impairment. MNA-SF was 13.0 (11.0–14.0) and 72.5% had normal nutritional status, 22.9% at risk and 4.6% malnutrition. Five participants died within a year.

**Table 1:** Characteristics of the participants

Demographic characteristics	Total	
	(*n* = 337)	
Age (years), Median (IQR)	78.0	(71.0–85.0)
Gender, n (%)		
Female	231	(68.5%)
Male	106	(31.5%)
Body height (cm), Median (IQR)	155.4	(151.0-161.3)
Body weight (kg), Median (IQR)	58.0	(50.8–64.8)
Body mass index (kg/m^2^), Median (IQR)	23.5	(21.0-25.9)
Educational level, n (%)		
Illiterate	59	(17.5%)
Literate	9	(2.7%)
Primary school	100	(29.7%)
Junior high school	49	(14.5%)
Senior high school	52	(15.4%)
University	68	(20.2%)
Marital Status, n (%)		
Single	4	(1.2%)
Married	172	(51.0%)
Widowed	153	(45.4%)
Divorced	8	(2.4%)
Living situation, n (%)		
Non-family/alone	55	(16.3%)
Family	282	(83.7%)
Smoking, n (%)	4	(1.2%)
Drinking, n (%)	2	(0.6%)
Betel nut chewing, n (%)	6	(1.8%)
**Comprehensive geriatric assessment**		
Charlson Comorbidity Index, Median (IQR)	2.0	(1.0–4.0)
Functional capacity		
Barthel index of activities of daily living, Median (IQR)	95.0	(85.0-100.0)
Lawton instrumental activities of daily living, Median (IQR)	6.0	(3.0–8.0)
Physical performance		
Handgrip strength, Median (IQR)	19.0	(14.6–24.3)
Low muscle strength, n (%)	198	(58.8%)
6-meter walking speed, Median (IQR)	0.9	(0.7–1.1)
Low physical performance, n (%)	170	(62.3%)
Timed Up and Go test (sec), Median (IQR)	12.0	(9.8–15.5)
Mini-mental state examination, Median (IQR)	26.0	(20.0–29.0)
Cognitive impairment, n (%)	96	(28.5%)
Five-item geriatric depression scale, Median (IQR)	1.0	(0.0–1.0)
Depressive symptoms, n (%)	80	(23.8%)
Mini-nutritional assessment-short form, Median (IQR)	13.0	(11.0–14.0)
Mini-nutritional assessment-short form, n (%)		
Normal	234	(72.5%)
At risk	74	(22.9%)
Malnutrition	15	(4.6%)
One-year mortality, n (%)	5	(1.5%)

The participants were divided into two groups by low hand grip strength (Table 2). In total, 139 (41.2%) participants were normal and 198 (58.8%) participants had possible sarcopenia. All parameters of CGA, chronic illness, functional capacity, physical performance, cognitive impairment, depressive symptoms, and malnutrition showed worse presentation in the possible sarcopenia group. There were no statistically significant differences in mortality between the two groups.

**Table 2:** Comparison between normal, and possible sarcopenia

Comprehensive geriatric assessment	Normal		Possible sarcopenia		*p* value
	(*n* = 139)		(*n* = 198)		
Charlson Comorbidity Index, Median (IQR)	2.0	(1.0–3.0)	3.0	(2.0–4.0)	0.019*
Functional capacity					
Barthel index of activities of daily living, Median (IQR)	100.0	(95.0-100.0)	90.0	(75.0-100.0)	< 0.001*
Lawton instrumental activities of daily living, Median (IQR)	7.0	(6.0–8.0)	5.0	(1.0–7.0)	< 0.001*
Physical performance					
Handgrip strength, Median (IQR)	23.7	(20.1–32.6)	15.2	(12.8–17.7)	< 0.001*
6-meter walking speed, Median (IQR)	1.0	(0.9–1.2)	0.9	(0.631.0)	< 0.001*
Low physical performance, n (%)	66	(49.6%)	104	(74.3%)	< 0.001*
Timed Up and Go test (sec), Median (IQR)	11.1	(9.0-14.5)	13.8	(10.8–16.7)	< 0.001*
Mini-mental state examination, Median (IQR)	28.0	(25.0–29.0)	23.0	(17.0–28.0)	< 0.001*
Cognitive impairment, n (%)	22	(15.8%)	74	(37.4%)	< 0.001*
Five-item geriatric depression scale, Median (IQR)	0.0	(0.0–1.0)	1.0	(0.0–2.0)	0.003*
Depressive symptoms, n (%)	24	(17.4%)	56	(28.3%)	0.021*
Mini-nutritional assessment-short form, Median (IQR)	14.0	(12.0–14.0)	12.0	(11.0–14.0)	< 0.001*
Mini-nutritional assessment-short form, n (%)					< 0.001*
Normal	116	(87.2%)	118	(62.1%)	
At risk	15	(11.3%)	59	(31.1%)	
Malnutrition	2	(1.5%)	13	(6.8%)	
One-year mortality, n (%)	0	(0.0%)	5	(2.5%)	0.080

*p < 0.05

As shown in Table 3, the participants were divided into three groups by BMD: normal, osteopenia, and osteoporosis. There were 23 (6.8%) normal participants, 124 (36.8%) participants with osteopenia and 190 (56.4%) participants with osteoporosis. HGS, physical performance, TUG, MMSE, and MNA-SF showed significant differences between these groups. In the post hoc analysis (data not shown), in the osteoporosis group, each of those variables showed the lowest values.

**Table 3:** Comparison between normal, osteopenia, and osteoporosis

Comprehensive geriatric assessment	Normal		Osteopenia		Osteoporosis		*p* value
	(*n* = 23)		(*n* = 124)		(*n* = 190)		
Charlson Comorbidity Index, Median (IQR)	3.0	(2.0–4.0)	2.0	(1.0–4.0)	2.0	(1.0–3.0)	0.064
Functional capacity							
Barthel index of activities of daily living, Median (IQR)	100.0	(90.0-100.0)	95.0	(90.0-100.0)	95.0	(85.0-100.0)	0.068
Lawton instrumental activities of daily living, Median (IQR)	7.0	(5.0–8.0)	7.0	(4.0–8.0)	6.0	(3.0–8.0)	0.164
Physical performance							
Handgrip strength, Median (IQR)	20.3	(15.0-32.1)	21.0	(16.8–27.5)	16.9	(13.5–22.1)	< 0.001*
Low muscle strength, n (%)	11	(47.8%)	58	(46.8%)	129	(67.9%)	0.001*
6-meter walking speed, Median (IQR)	1.0	(0.9–1.2)	1.0	(0.7–1.2)	0.9	(0.7–1.1)	0.182
Low physical performance, n (%)	8	(40.0%)	57	(55.3%)	105	(70.0%)	0.006*
Timed Up and Go test (sec), Median (IQR)	9.9	(8.6–13.2)	11.8	(9.2–15.0)	12.7	(10.5–16.2)	0.009*
Mini-mental state examination, Median (IQR)	28.0	(24.0–29.0)	27.0	(23.0–29.0)	25.0	(19.0–29.0)	0.005*
Cognitive impairment, n (%)	5	(21.7%)	33	(26.6%)	58	(30.5%)	0.572
Five-item geriatric depression scale, Median (IQR)	0.0	(0.0–1.0)	1.0	(0.0–1.0)	1.0	(0.0–2.0)	0.552
Depressive symptoms, n (%)	4	(17.4%)	27	(22.0%)	49	(25.8%)	0.558
Mini-nutritional assessment-short form, Median (IQR)	14.0	(12.0–14.0)	13.0	(12.0–14.0)	13.0	(11.0–14.0)	0.012*
Mini-nutritional assessment-short form, n (%)							0.399
Normal	19	(82.6%)	91	(75.8%)	124	(68.9%)	
At risk	4	(17.4%)	25	(20.8%)	45	(25.0%)	
Malnutrition	0	(0.0%)	4	(3.4%)	11	(6.1%)	
One-year mortality, n (%)	0	(0.0%)	1	(0.8%)	4	(2.1%)	0.756

*p < 0.05

According to the clinical definition of osteosarcopenia, 78 (23.1%) participants were normal, 69 (20.5%) participants showed possible sarcopenia based on HGS, 276 (81.9%) participants were normal/osteopenia and 61 (18.1%) participants had osteoporosis according to BMD. Therefore, 129 (38.3%) participants were considered to have possible osteosarcopenia (Table 4). Among the 4 groups, statistically significant differences in the number of chronic illnesses, ADL, 6MWS, cognitive impairment, and malnutrition were observed. The post hoc analysis revealed that these differences persisted between the possible osteosarcopenia group and normal group. Moreover, daily activities, walking speed, cognitive score and nutrition score were found to be significantly lower in the possible osteosarcopenia group in comparison with the osteoporosis group. However, there were no significant differences between the possible osteosarcopenia group and the possible sarcopenia only (data not shown).

**Table 4:** Comparison between normal, osteoporosis, possible sarcopenia, possible osteosarcopenia

Comprehensive geriatric assessment	Normal		only Osteoporosis		only Possible sarcopenia		Osteoporosis with possible sarcopenia		*p* value
	(*n* = 78)		(*n* = 61)		(*n* = 69)		(*n* = 129)		
Charlson Comorbidity Index, Median (IQR)	2.0	(1.0–3.0)	2.0	(1.0–3.0)	3.0	(2.0–5.0)	2.0	(1.0–3.0)	< 0.001*
Functional capacity									
Barthel index of activities of daily living, Median (IQR)	100.0	(95.0-100.0)	100.0	(95.0-100.0)	90.0	(82.5–100.0)	90.0	(75.0-100.0)	< 0.001*
Lawton instrumental activities of daily living, Median (IQR)	7.0	(6.0–8.0)	7.0	(5.0–8.0)	5.0	(1.0–7.0)	5.0	(1.5-7.0)	< 0.001*
Physical performance									
Handgrip strength, Median (IQR)	25.3	(20.6–33.9)	22.2	(19.9–26.8)	16.7	(13.9–20.8)	14.6	(12.4–17.0)	< 0.001*
6-meter walking speed, Median (IQR)	1.1	(0.9–1.2)	0.9	(0.8–1.2)	0.8	(0.6-1.0)	0.9	(0.7-1.0)	< 0.001*
Low physical performance, n (%)	28	(37.8%)	38	(64.4%)	37	(75.5%)	67	(73.6%)	< 0.001*
Timed Up and Go test (sec), Median (IQR)	10.1	(8.4–13.3)	12.6	(10.1–15.7)	14.2	(10.8–16.4)	12.9	(10.7–16.9)	< 0.001*
Mini-mental state examination, Median (IQR)	28.0	(26.0–29.0)	27.0	(23.5–29.0)	24.0	(17.5–28.0)	23.0	(17.0–28.0)	< 0.001*
Cognitive impairment, n (%)	10	(12.8%)	12	(19.7%)	28	(40.6%)	46	(35.7%)	< 0.001*
Five-item geriatric depression scale, Median (IQR)	1.0	(0.0–1.0)	0.0	(0.0–1.0)	1.0	(0.0–2.0)	1.0	(0.0–2.0)	0.022*
Depressive symptoms, n (%)	13	(16.9%)	11	(18.0%)	18	(26.1%)	38	(29.5%)	0.131
Mini-nutritional assessment-short form, Median (IQR)	14.0	(13.0–14.0)	13.0	(12.0–14.0)	12.0	(11.0–14.0)	13.0	(11.0–14.0)	< 0.001*
Mini-nutritional assessment-short form, n (%)									< 0.001*
Normal	68	(90.7%)	48	(82.8%)	42	(61.8%)	76	(62.3%)	
At risk	6	(8.0%)	9	(15.5%)	23	(33.8%)	36	(29.5%)	
Malnutrition	1	(1.3%)	1	(1.7%)	3	(4.4%)	10	(8.2%)	
One-year mortality, n (%)	0	(0.0%)	0	(0.0%)	1	(1.5%)	4	(3.1%)	0.322

*p < 0.05

To determine the relationship between osteoporosis, possible sarcopenia, ADL disability, cognitive impairment, and malnutrition, we found that possible osteosarcopenia was associated with ADL disability and cognitive impairment in the univariable analysis. After adjustment for the other potential confounders, there remained significant association between possible osteosarcopenia and cognitive impairment (Table 5).

**Table 5:** Predictors of decline of activities of daily living, cognitive impairment, malnutrition

	Decline of activities of daily living								Cognitive impairment								Malnutrition (normal/at risk vs. malnutrition)							
	Univariate				Multivariate				Univariate				Multivariate				Univariate				Multivariate			
	**OR**	**95%CI**		***p value***	**OR**	**95%CI**		***p value***	**OR**	**95%CI**		***p value***	**OR**	**95%CI**		***p value***	**OR**	**95%CI**		***p value***	**OR**	**95%CI**		***p value***
Age (years)	1.12	(1.06-	1.19)	< 0.001*	0.99	(0.87-	1.13)	0.880	1.10	(1.07-	1.14)	< 0.001*	1.00	(0.94-	1.06)	0.889	1.03	(0.96-	1.10)	0.400				
Gender																								
Female	1.00								1.00								1.00							
Male	0.60	(0.23-	1.53)	0.286					0.99	(0.59-	1.64)	0.959					0.15	(0.02-	1.13)	0.065				
Osteosarcopenia																								
Normal	1.00				1.00				1.00				1.00				1.00							
only Osteoporosis	0.00	(0.00-		0.997	0.00	(0.00-		0.996	1.67	(0.67-	4.16)	0.275	1.94	(0.54-	7.03)	0.311	1.30	(0.08-	21.21)	0.855				
only Possible sarcopenia	7.33	(0.86-	62.52)	0.068	0.14	(0.01-	2.53)	0.182	4.64	(2.05-	10.54)	< 0.001*	2.45	(0.65-	9.17)	0.183	3.42	(0.35-	33.64)	0.293				
Osteoporosis with possible sarcopenia	14.13	(1.86-	107.52)	0.011*	1.05	(0.07-	16.32)	0.974	3.77	(1.77-	8.02)	0.001*	3.67	(1.10-	12.29)	0.035*	6.61	(0.83-	52.70)	0.075				
Comprehensive geriatric assessment																								
Charlson Comorbidity Index	1.23	(1.002-	1.51)	0.048*	1.12	(0.77-	1.63)	0.555	1.23	(1.08-	1.41)	0.002*	0.96	(0.76-	1.22)	0.742	0.80	(0.57-	1.12)	0.190				
Barthel index of activities of daily living									0.95	(0.93-	0.97)	< 0.001*	1.05	(0.99-	1.12)	0.118	0.96	(0.95-	0.98)	< 0.001*	0.98	(0.95-	1.01)	0.146
Lawton instrumental activities of daily living	0.21	(0.11-	0.40)	< 0.001*	0.16	(0.06-	0.40)	< 0.001*	0.64	(0.58-	0.71)	< 0.001*	0.67	(0.52-	0.85)	0.001*	0.70	(0.57-	0.85)	< 0.001*	0.90	(0.66-	1.24)	0.535
Physical performance																								
Low physical performance									2.73	(1.37-	5.45)	0.004*	0.91	(0.31-	2.63)	0.860	0.93	(0.22-	3.99)	0.924				
Timed Up and Go test	1.11	(0.99-	1.23)	0.071					1.12	(1.06-	1.19)	< 0.001*	1.08	(0.98-	1.18)	0.110	1.07	(0.99-	1.15)	0.096				
Cognitive impairment	8.80	(3.58-	21.61)	< 0.001*	1.77	(0.44-	7.19)	0.425									7.71	(2.39-	24.88)	0.001*	3.69	(0.97-	14.12)	0.056
Depressive symptoms	1.13	(0.46-	2.78)	0.788					1.49	(0.87-	2.55)	0.146					3.02	(1.06-	8.62)	0.039*	2.91	(0.95-	8.89)	0.061
Mini-nutritional assessment-short form																								
Normal	1.00				1.00				1.00				1.00											
At risk	3.17	(1.29-	7.79)	0.012*	1.27	(0.35-	4.60)	0.713	2.65	(1.52-	4.63)	0.001*	1.00	(0.39-	2.55)	0.997								
Malnutrition	13.52	(4.08-	44.75)	< 0.001*	6.06	(0.77-	47.48)	0.086	10.12	(3.09-	33.14)	< 0.001*	2.99	(0.48-	18.65)	0.240								

*p < 0.05. After adjustment for those variables significantly related to dichotomous outcomes in the univariate analysis.

## Discussion

Osteosarcopenia, a relatively new geriatric syndrome, is defined as the coexistence of both osteoporosis and sarcopenia. In recent years, the condition has gained more attention due to its potential to become a substantial global health challenge. Our study found that in community-dwelling older adults, 78 participants were normal, 69 participants showed possible sarcopenia, based on HGS, whereas 146 participants were normal/osteopenia and 190 participants had osteoporosis, based on BMD. Older adults with possible osteosarcopenia had the highest prevalence of comorbidities, cognitive, daily activity impairment, and malnutrition. Moreover, possible osteosarcopenia was more strongly associated with cognitive impairment than osteoporosis or sarcopenia alone.

The criteria for a definitive clinical diagnosis of sarcopenia are based on both muscle strength and mass measured by DEXA or BIA according to the AWGS 2019 guidelines [11]. Of particular note, the guidelines introduced the concept of possible sarcopenia with the presence of either low muscle strength or low physical performance alone. The guidelines are intended for use in primary healthcare or community-based health promotion settings to facilitate earlier identification and lifestyle interventions, notably in vulnerable older patients with limited access to healthcare services [2]. Although there is ongoing debate about the appropriateness of using HGS to assess global or lower limb muscle function, it is worth noting that HGS is recommended as the primary screening test for sarcopenia in international guidelines [22]. In our study, we identified possible sarcopenia using HGS and categorized the surveyed community participants accordingly. Among the participants, 198 (58.8%) individuals were identified as having possible sarcopenia, and 129 (38.3%) individuals exhibited possible osteosarcopenia. A previous study indicated that the prevalence of sarcopenia in Taiwan may affect about 10% of individuals aged 60 to 70, and increased to about 30% among those aged 80 and above [23]. Furthermore, another study conducted in Southeast Asian population in Singapore revealed that the prevalence of osteosarcopenia was 17.3% in individuals aged 65 years and older, and increased to 25.5% among those aged 75 years and older [24]. In our study, the prevalence remained relatively high, which may be attributed to biological and lifestyle factors, as well as the variation in diagnostic methods and operational definitions.

Bone and muscle are linked not just physically but also chemically and metabolically. Shared pathophysiological features like fat infiltration and altered stem cell differentiation further underscore the close relationship between sarcopenia and osteoporosis [13]. Therefore, osteosarcopenia has gained more attention in recent years due to its association with adverse outcomes, including increased risk of falls, fractures, functional impairment, frailty, and even mortality [1, 7, 12, 13, 25]. In our study, we found that mortality was not different between the possible osteosarcopenia group and the other groups, which was consistent with a previous study [26]. However, possible osteosarcopenia was more strongly associated with disease burden, impaired daily activities, cognitive impairment, and malnutritional status.

In older adults, osteosarcopenia was associated with declines in physical function, mobility, and balance, which could lead to limitations in ADLs and IADLs and ultimately contribute to disability [27]. Furthermore, osteosarcopenia could increase the risk of frailty, a condition characterized by decreased reserve capacity and increased vulnerability to stressors, which could also lead to disability [28]. Our study found evidence that possible osteosarcopenia had poorer physical function than the other groups, although a significant ADL disability risk could not be demonstrated in the regression analysis. It has been proposed that shared mechanisms exist between osteoporosis and sarcopenia, such as hormonal changes and inflammation, affecting both bone and muscle health. Extensive research has demonstrated that sarcopenia directly influences muscle function, leading to decreased strength, balance, and functional capacity. Due to the similarities between osteoporosis and sarcopenia, osteosarcopenia, which involves a compromised musculoskeletal system, amplifies the vulnerability to falls, fractures, and overall mobility constraints [27].

In a meta-analysis, sarcopenia was associated with cognitive impairment [29], and similarly, osteoporosis was associated with cognitive impairment [30]. The former study showed osteosarcopenia was more closely related to physical and cognitive dependence, frailty, and death [31]. Interestingly, a recent study showed osteosarcopenia was independently associated with cognitive frailty and it had a higher odds ratio than osteoporosis or sarcopenia alone [32, 33]. In our study, osteoporosis with possible sarcopenia was associated with cognitive impairment in older adults. In the literature, a number of factors have been found to influence the development of cognitive impairment and osteosarcopenia, including excessive oxidative stress, diminished physical activity, inadequate dietary intake, and age-related chronic low-grade inflammation. Notably, excessive oxidative stress can trigger cellular dysfunction and harm, ultimately leading to cognitive decline and the distinctive musculoskeletal degeneration inherent in osteosarcopenia. Furthermore, age-related chronic low-grade inflammation, a shared hallmark of both conditions, can disrupt crucial physiological processes, exacerbating cognitive impairment and contributing to the multifaceted degenerative processes inherent to osteosarcopenia [29, 34, 35].

Nutritional status can be affected by various factors, including dietary intake, nutrient absorption and metabolism, individual health conditions, and socioeconomic factors. It can be evaluated using a number of indicators and assessment methods, and therefore, it is important to note that the treatment of malnutrition requires a multidisciplinary approach involving healthcare professionals, nutritionists, policymakers, and community support systems [36]. Malnutrition could be related to osteosarcopenia, as adequate nutrition is essential for maintaining bone and muscle health. In line with a previous study as well as ours [37], poor nutritional status was found to be associated with possible osteosarcopenia. Osteosarcopenia, which affects muscle and bone, can lead to reduced appetite and mobility, thereby worsening malnutrition. Moreover, malnutrition can exacerbate osteosarcopenia by contributing to muscle and bone loss, which in turn impair function [1, 8, 37]. Both osteosarcopenia and sarcopenia underscore the importance of holistic care. Addressing malnutrition involves not only nutritional interventions, but also consideration of the broader physical, psychological, and social dimensions.

A previous study showed that compared with the nonosteosarcopenic group, those with osteosarcopenia had greater impairment of physical performance and balance. In that study, sarcopenia was defined as low appendicular lean mass plus low muscle strength or low physical performance [38]. Another study had reported that co-occurrence osteosarcopenia was frequent and it was associated with a more compromised nutritional state than isolated osteoporosis or sarcopenia [39]. In this study, we found that the combination of osteoporosis and possible sarcopenia was associated with an increased risk of cognitive function impairment but not with ADL dysfunction and malnutrition. The reasons for the inconsistent findings compared to previous studies were not entirely clear. It is speculated that in our study’s participants, factors other than osteoporosis and sarcopenia may have a more significant impact on driving physical dysfunction and malnutrition. Besides, it was worthwhile to consider the other limitations in our study, such as sample size, the method to measure sarcopenia, and the selected cutoff values for ADL disability. On the contrary, previous reports as well as ours showed a closer bidirectional relationship between musculoskeletal health and cognitive function [40], although the exact causal relationship remained to be elucidated.

In our study, the possible osteosarcopenia group showed poorer GDS-5 scores, although there was no significant difference in depressive mood. Differing from prior research, it has been noted that depressive mood tends to be higher in cases of osteosarcopenia compared to osteoporosis or sarcopenia alone [41]. Some studies reported osteosarcopenia was often linked to symptoms of depression, which may be due to the negative impact of physical health on mental health. In addition, depression might also cause a decline in physical function, thereby exacerbating the symptoms of osteosarcopenia [14, 41]. Declining physical function due to bone and muscle loss may lead to social isolation and reduced activity, contributing to depression. Conversely, depression affects physical activity, hormone levels, and bone metabolism, potentially worsening osteoporosis and sarcopenia. This bidirectional connection forms a complex cycle of interactions [1, 8]. In conclusion, the interplay among osteosarcopenia, sarcopenia, and depressive mood is complex and therefore necessitates a nuanced approach.

Osteoporosis and sarcopenia are chronic and progressive conditions. Fragmented care models that address bone health and sarcopenia separately have limitations. Hence, the comprehensive geriatric assessment holds value for community-dwelling older adults. An essential component of osteosarcopenia follow-up involves identifying and directing high-risk individuals to specialized multidisciplinary clinics.

There were several limitations in this study. First, we did not verify the diagnosis of sarcopenia, which might have led to selection bias and influenced outcomes. Second, the study did not analyze data on the patients’ drug prescriptions and laboratory examinations, both of which could have had a significant impact on physical and mental functions. Third, the findings were based on data from a single medical center, which means that the findings may not be generalizable to other populations. However, the study result may be more suitable for the individuals receiving non-imaging evaluation for sarcopenia. Finally, further longitudinal analyses with larger numbers of participants are required to establish whether osteosarcopenia is causally linked with cognition and physical functions, as well as nutritional status in community-dwelling older adults.

## Conclusions

This study found that worse daily activity, walking speed, cognitive impairment, and malnutrition were associated with osteoporosis with possible sarcopenia. Moreover, osteosarcopenia might significantly increase the risk of cognitive impairment, as measured by MMSE. In consideration of the association between osteosarcopenia and cognitive impairment, earlier screening of and interventional strategies for cognitive function in older adults with possible osteosarcopenia may improve their health outcomes, although the causal relationship between osteosarcopenia and cognitive impairment requires further study.

## Data Availability

The datasets used and analyzed during the current study are available from the corresponding author on reasonable request.

## Abbreviations

6MWS: 6-meter walking speed
ADL: Activities of daily living
BI: Barthel index
BIA: Bioelectrical impedance analysis
BMI: Bone mineral density
CGA: Comprehensive geriatric assessment
DEXA: Dual-energy X-ray absorptiometry
GDS-5: Five-item geriatric depression scale
HGS: Handgrip strength
IADL: Instrumental activities of daily living
IQR: Interquartile range
MMSE: Mini-mental state examination
MNA-SF: Mini-nutritional assessment-short form
TUG: Timed Up and Go
WHO: World Health Organization
